# Long-term exposure to fine particulate matter and ozone and the onset of systemic autoimmune rheumatic diseases: an open cohort study in Quebec, Canada

**DOI:** 10.1186/s13075-022-02843-5

**Published:** 2022-06-23

**Authors:** Naizhuo Zhao, Audrey Smargiassi, Sonia Jean, Philippe Gamache, Elhadji-Anassour Laouan-Sidi, Hong Chen, Mark S. Goldberg, Sasha Bernatsky

**Affiliations:** 1 Division of Clinical Epidemiology, McGill University Health Centre, Montreal, QC Canada; 2 Département de Santé Environnementale Et de Santé Au Travail, School of Public Health, Université de Montréal, Montréal, QC Canada; 3 Institut National de Santé Publique du Québec, Montréal, QC Canada; 4 Centre of Public Health Research, University of Montreal and CIUSSS du Centre-Sud-de-L’Île-de-Montréal, Montreal, Canada; 5 Université Laval and Bureau d’information Et d’études en Santé Des Populations, Institut National de Santé Publique du Québec (INSPQ), 945, avenue Wolfe, Québec, QC G1V 5B3 Canada; 6 Environmental Health Science and Research Bureau, Health Canada, Ottawa, ON Canada; 7 Institute for Clinical Evaluative Sciences, Toronto, ON Canada; 8 Public Health Ontario, Toronto, ON Canada; 9 Dalla Lana School of Public Health, University of Toronto, Toronto, ON Canada; 10 Department of Medicine, McGill University, Québec, Canada; 11 Divisions of Rheumatology and Clinical Epidemiology, McGill University Health Centre, Montreal, QC Canada; 12 Centre for Outcomes Research & Evaluation, Research Institute of the McGill University Health Centre, 5252 Boul. de Maisonneuve Ouest, (3F.51), Montreal, QC H4A 3S5 Canada

**Keywords:** Systemic autoimmune rheumatic diseases, Air pollution, Fine particulate matter, Ozone

## Abstract

**Objectives:**

To estimate associations between fine particulate matter (PM_2.5_) and ozone and the onset of systemic autoimmune rheumatic diseases (SARDs).

**Methods:**

An open cohort of over 6 million adults was constructed from provincial physician billing and hospitalization records between 2000 and 2013. We defined incident SARD cases (SLE, Sjogren’s syndrome, scleroderma, polymyositis, dermatomyositis, polyarteritis nodosa and related conditions, polymyalgia rheumatic, other necrotizing vasculopathies, and undifferentiated connective tissue disease) based on at least two relevant billing diagnostic codes (within 2 years, with at least 1 billing from a rheumatologist), or at least one relevant hospitalization diagnostic code. Estimated PM_2.5_ and ozone concentrations (derived from remote sensing and/or chemical transport models) were assigned to subjects based on residential postal codes, updated throughout follow-up. Cox proportional hazards models with annual exposure levels were used to calculate hazard ratios (HRs) for SARDs incidence, adjusting for sex, age, urban-versus-rural residence, and socioeconomic status.

**Results:**

The adjusted HR for SARDS related to one interquartile range increase in PM_2.5_ (3.97 µg/m^3^) was 1.12 (95% confidence interval 1.08–1.15), but there was no clear association with ozone. Indirectly controlling for smoking did not alter the findings.

**Conclusions:**

We found associations between SARDs incidence and PM_2.5_, but no relationships with ozone. Additional studies are needed to better understand interplays between the many constituents of air pollution and rheumatic diseases.

**Supplementary Information:**

The online version contains supplementary material available at 10.1186/s13075-022-02843-5.

## Introduction

Ambient air pollution is the fifth leading cause of deaths worldwide [1]. Exposure to particulate matter, especially fine particles of diameter 2.5 µm or less (PM_2.5_), increases risk of developing or dying from cardiac, cerebrovascular, and chronic airway diseases [2]. Only a few studies have investigated the effects of air pollution on rheumatic diseases [3], and the evidence of an association from these studies is not persuasive [4].

Systemic autoimmune rheumatic diseases (SARDs), including systemic lupus erythematosus (SLE) and related conditions, are complex chronic rheumatic disorders that cause high personal and societal burdens [5]. The etiology of SARDs is poorly understood. Inhaled air pollutants (e.g., PM_2.5_ and ozone) can increase oxidative stress and inflammatory responses in the lung and potentially trigger systemic immune system changes [6]. However, to date, no studies of air pollution and the incidence of SARDs have been published. Our purpose was to determine whether the incidence of SARDs is associated with exposures to ambient PM_2.5_ and ozone.

## Methods

We used provincial administrative health data from the province of Quebec (population of approximately 8.2 million in 2013) to create this population-based cohort [7]. In Canada, residents of each province are recipients of provincial universal health care plans, covering physician encounters (including all clinic, inpatient, and emergency room encounters) and hospitalizations. Information about these encounters (with International Classification of Diseases, ICD, billing claim diagnoses for physician encounters, and primary and up to 25 non-primary ICD diagnostic codes for hospitalizations) is maintained in provincial administrative databases. From these databases, we formed an open cohort initially of all Quebec residents who were 18 years of age and older as of April 1, 2000. Prior to cohort entry, subjects were required to have lived in the province of Quebec for at least 4 years and to be free of SARDs, based on no physician billings or hospitalizations. After 2000, residents who met these entry criteria were added to the cohort in each subsequent calendar year. Follow-up extended from cohort until the first of date of SARDs diagnosis, death, migration from the province, or end of study (i.e., December 31, 2012). Information on age, sex, six-character residential postal codes throughout the entire follow-up, and physician visits and hospitalizations was available for all subjects.

An incident SARD case was defined on the basis of two or more physician billing claims with a relevant ICD diagnostic code within 2 years, including at least one relevant physician claim from a rheumatologist, or one or more hospitalizations with a relevant ICD diagnostic code. The codes of relevance included ICD-9 446, 710, or 725 and ICD-10 diagnostic codes of M30, M31, M32.1, M32.8, M32.9, M33, M34, M35.0, M35.3, M35.5, or M35.9. These diagnostic codes include SLE, Sjogren’s syndrome, scleroderma, polymyositis, dermatomyositis, polyarteritis nodosa and related conditions, polymyalgia rheumatic, other necrotizing vasculopathies, and undifferentiated connective tissue disease. This approach to rheumatic disease case definition has been adapted for use within the Public Health Agency of Canada’s national chronic disease surveillance system, and our previous work has estimated high specificity and sensitivity for the use of administrative claims data for SARDs diagnoses in Quebec [8].

We used estimates of concentrations of PM_2.5_ for 2000–2012 derived from complex models including the GEOS (Goddard Earth Observing System) Chemical Transport Model (GEOS-Chem) and remotely sensed measurements of aerosol optical depth. GEOS-Chem (http://geos-chem.org) is a 3-D model simulating atmospheric chemistry by meteorological factors. These relatively coarse PM_2.5_ image products (at the 10 km resolution) were further resampled to a 1-km (km) spatial resolution using geographically weighted regression modeling. The PM_2.5_ estimates hold a high agreement (*R*^2^ = 0.81) with PM_2.5_ concentrations from ground monitoring stations [9]. Hourly concentrations of ozone simulated by the Canadian Hemispheric and Regional Ozone and NO_x_ System Chemical Transport Model and measured by ground monitoring stations were combined using an optimal interpolation scheme [10]. The combined ozone concentrations have been further averaged for each calendar year between 2002 and 2012 and exported as a gridded dataset at a spatial resolution of 21 km. Ozone data for 2002 was used for 2000 and 2001. We assigned for the days of follow-up of each subject, annual average daily concentrations of PM_2.5_, and ozone at their residential address according to their postal code of residence. Thus, a person followed for the 13-year period of observation (i.e., 2000–2012) and residing at the same location for that period would have the same yearly exposure average applied for all days of each year. If a person moved within a year, two different exposure values were assigned, one for the days at the old address and one for the days at the new address.

Using a 10% random sample and the Bayesian approach developed by Nasari et al. [11], we assessed possible linear and nonlinear relationships between SARDs onset and exposures to the two individual air pollutants. This preliminary assessment showed that linear functions described the relationships best (i.e., with the lowest Akaike information criterion). Given the Bayesian approach is rather inefficient in terms of computing time, we used Cox proportional hazards models with time-varying exposure levels instead of the Bayesian approach for all individuals followed in the cohort. Multi-variable Cox proportional hazards models for time to SARD onset were developed separately for each of the two continuous variables of the air pollutants, adjusting for age and sex with time since cohort entry as the time axis. We did not combine PM2.5 and ozone in the same models because air pollution concentrations are closely correlated with each other [12]. Previous data suggest that socioeconomic variables may also influence risk of SARDs, and thus, we adjusted for sex, age, and socioeconomic status (SES) at cohort entry. SES was estimated using the Pampalon material deprivation index (indicating lack of financial resources) [13], from the least (Quintile-1) to the most (Quintile-5) deprived (see Table 1 for the detailed categories). These index values are assigned at the level of dissemination area, which consists of one or more neighboring blocks of houses with a population of 400 to 700. Since regional variations in SES may potentially confound associations between disease onset and air pollution [3], we adjusted for sex, age, socioeconomic status, and urban-versus-rural residence in another group of models. To classify urban-versus-rural residence, we used the Statistics Canada classification system to define major census metropolitan areas (CMA), other CMAs, census agglomerations, and rural areas. Given that women have a higher incidence of SARDs [14] and that air pollution has been shown to influence outcomes differently in men and women [15], we performed a sensitivity analysis stratifying by sex.

**Table 1:** Distribution of selected characteristics of the entire cohort, Quebec, 2000–2013

Variable		Number at cohort entry (percentage)	Pearson-years of observation
**Age**^a^	20–44	3,646,390 (57.1)	28,548,549
	45–64	1,794,103 (28.1)	25,477,943
	65 +	942,017 (14.8)	13,484,923
**Sex**	Male	3,108,864 (48.7)	32,380,859
	Female	3,273,646 (51.3)	35,730,556
**Material deprivation**	Quintile 1	1,244,647 (19.5)	12,952,185
	Quintile 2	1,241,466 (19.4)	13,214,565
	Quintile 3	1,267,927 (19.9)	13,510,431
	Quintile 4	1,258,677 (19.7)	13,219,538
	Quintile 5	1,230,893 (19.3)	12,606,293
	Missing	138,900 (2.2)	2,008,403
**Urban–rural residence**	Rural	1,378,184 (21.6)	31,108,277
	Major CMA^a^ (Montreal)	2,969,913 (46.5)	13,420,259
	Other CMAs	1,238,883 (19.4)	8,581,158
	Census Agglomeration	795,530 (12.5)	14,401,721

^a^We split age into the three categories to facilitate comparison with the previous studies [3, 16]. CMA census metropolitan area

Previous studies have suggested that smoking may increase risks of certain SARDs (e.g., SLE) [17], and it is possible that local smoking habits may vary systematically with regional variations in air pollution levels. However, smoking information is not collected in Canada’s administrative health data. Thus, as others have done [18], we performed an additional indirect adjustment of our HRs for smoking. The method requires information on linear associations between the variables included in the survival models and the variable indirectly adjusted for (i.e., smoking). To derive these associations, we used data on the percentage of Quebec residents living in postal code regions who were current/former vs. never smokers, based on the 2005–2015 Canadian Community Health Survey (CCHS) and the published HR for current/former smoking and SARDs onset [17]. Because ozone data has not been linked to CCHS smoking data, we did not conduct this indirect adjustment for the analyses of ozone and SARDs.

## Results

After excluding 294,667 individuals with missing data (4.6%), we followed 6,104,859 adults (51.4% female) without SARDs for a total of 63,042,096 person-years (average 10 years), with 32,179 new SARDs cases during this time (5.1 cases per 10,000 person-years). Among the 32,179 new SARDs patients, diffuse diseases of connective tissue and polymyalgia rheumatica were the most frequent (35.1% and 25.5% of cases respectively these codes). The number of cases defined by each ICD diagnostic code is shown in Table S1. Statistics on the entire cohort are shown in Table 1. The average PM_2.5_ and ozone concentrations across Quebec were 7.5 (standard deviation, SD 2.5) µg/m^3^ and 24.0 (SD 4.1) parts per billion (ppb) respectively. The interquartile ranges of PM_2.5_ and ozone were 4.0 µg/m^3^ and 5.6 ppb respectively. The annual average PM_2.5_ concentrations for Quebec decreased steadily from 8.9 to 6.6 µg/m^3^ during 2000 to 2012 while the annual ambient ozone concentration increased from 21.6 to 31.8 ppb. The average exposure of PM_2.5_ was weakly correlated to that of ozone with a Spearman correlation coefficient of 0.22 (*p* < 0.01). More detailed distribution of population weighted PM_2.5_ and ozone exposures are shown in Table S2.

Table 2 shows the adjusted HR and 95% confidence intervals (CIs) for the linear associations between the two air pollutants and SARDs incidence. With the overall subjects in our sample, the adjusted HRs suggested a positive association of SARD onset with PM_2.5_ exposure, but no clear associations were found for ozone. Similar results were observed in the male and female subgroups. After additional indirect adjustment for smoking, the adjusted HR for the association between SARDs and PM_2.5_ was not greatly change (see Table 2).

**Table 2:** Adjusted HRs (95% CIs) from Cox proportional hazards models for the linear association between SARDs and per interquartile increment in PM_2.5_ or ozone exposure.^a^, adjusting for different covariates, Quebec, 2000–2013

Covariates included in the model	PM_2.5_	Ozone
Age and sex	1.25 (1.16–1.34)	0.96 (0.82–1.00)
Age, sex, and deprivation index	1.22 (1.13–1.31)	0.99 (0.94–1.03)
Age, sex, deprivation index, and urban–rural residence	1.22 (1.11–1.35)	0.96 (0.89–1.02)
Age, sex, deprivation index, urban–rural residence, and smoking^b^	1.22 (1.10–1.34)	-
Age, deprivation index, and urban–rural residence (male subgroup)	1.29 (1.08–1.55)	1.05 (0.95–1.15)
Age, deprivation index, and urban–rural residence (female subgroup)	1.20 (1.06–1.35)	0.97 (0.98–1.00)

^a^The interquartile ranges of PM_2.5_ and ozone are 3.97 µg/m.^3^ and 5.55 ppb respectively

^b^ Indirect adjustment based on ancillary data from the Canadian Community Health Survey

## Discussion

Our findings of a positive association between PM_2.5_ exposure and SARDs incidence are consistent with our previous work using prevalent SARD diagnoses in Quebec and in Alberta [3, 16]. Ours is the first population-based study of incident SARDs and air pollution. We did not find a clear association between ozone and SARD onset. No one has previously studied ozone and SARD onset, although a positive association between ozone and rheumatoid arthritis onset was observed in one study from British Columbia, Canada [19]. However, we used more precise methods to estimate ozone exposure.

The use of a large population-based sample is a great strength of our study. Also, we evaluated incident disease, in contrast to previous work using prevalent cases [16]. Moreover, in our previous studies [3, 16], each subject was assigned a fixed exposure value. In our study, we used time-varying air pollutants levels for the entire period of follow up of the individuals to better reflect exposures over time and fully consider the residential mobility.

We should note that, in using administrative health data, we are only studying individuals who have presented for (and received) health care. However, SARDs are most often symptomatic (thus patients are likely to present for care), and our previous analyses have suggested that an average of 10 years of follow-up is adequate to detect SLE, one of the more common SARDs [20].

As most existing studies did, we assigned to each participant concentrations of PM_2.5_ and ozone at the central point of the postal code of his/her residence. However, people are mobile in their cities for different daily activities while locations of those activities cannot be fully recorded [21]. Thus, we admit that some exposure misclassifications result from the current assignment method. Additionally, a postal code in a rural region tends to cover a larger area of land than that in an urban region. However, we do not believe this leads to important misclassification of exposures. Variance in air pollutant concentrations in rural areas is relatively small, even though a rural participant’s house and the central point of the postal code of the participant within a region may be relatively far away.

The average PM_2.5_ and ozone concentrations across Quebec are lower than the standard air pollutant levels of 8.8 µg/m^3^ and 62 ppb (for 8-h average) respectively defined by the Canadian Ambient Air Quality Standards (CAAQS) [22]. In fact, health effects below the CAAQS standards have been demonstrated in many previous studies [23, 24].

Not having exposure data prior to entering the cohort is a limitation of this study. We do acknowledge the relatively coarse spatial resolution of the ozone data, which could have masked a true significant association between ozone and SARDs onset. Moreover, the significance of the relationship between ozone exposure and SARDs may be different after adding more covariates such as individual smoking habit to the model. Ambient ozone levels have great seasonal variations and ozone levels may spike to exceptionally high a few days each year. Although we did not observe a clear association between long-term exposure to ozone and SARDs, we did not model effects of short-term very high ozone levels on SARDs. Also, our analyses do no preclude the possibility that jurisdictions with higher ozone concentrations might yield different results.

## Conclusions

PM_2.5_ exposures were associated with higher risks of SARDs onset in Quebec. Indirectly controlling for smoking did not alter the association. We did not observe conclusive associations between SARDs onset and ozone exposure in Quebec. However, this does not preclude the possibility that ozone concentrations might be associated with rheumatic disease in other settings or jurisdictions. Additional studies are needed to better understand interplays between the many constituents of air pollution and rheumatic disease.

## Supplementary Information

**Additional file 1:** **Table S1.** International Classification of Diseases (ICD) diagnostic codes for defining systemic autoimmune rheumatic diseases cases and their corresponding numbers of cases. **Table S2.** Distribution of population weighted concentrations of ambient PM2.5 and ozone in Quebec. SD: standard deviation.

## Data Availability

The dataset used and analyzed during the current study is available from Institut National de Santé Publique du Québec (INSPQ) on reasonable request.

## Abbreviations

CAAQS: Canadian Ambient Air Quality Standards
CCHS: Canadian Community Health Survey
CI: Confidence interval
CMA: Census metropolitan areas
GEOS-Chem: Goddard Earth Observing System Chemical Transport Model
ICD: International Classification of Diseases
HR: Hazard ratios
PM_2.5_: Fine particulate matter
SARDs: Systemic autoimmune rheumatic diseases
SES: Socioeconomic status
SD: Standard deviation
SLE: Systemic lupus erythematosus
