# Supplementary material for: Long-term exposure to fine particulate matter and ozone and the onset of systemic autoimmune rheumatic diseases: an open cohort study in Quebec, Canada
Source: Arthritis Res Ther. 2022 Jun 23;24:151. doi: 10.1186/s13075-022-02843-5 (PMC9219240; doi:10.1186/s13075-022-02843-5)
Supplement: Supplementary file 1 — Additional file 1: Table S1. International Classification of Diseases (ICD) diagnostic codes for defining systemic autoimmune rheumatic diseases cases and their corresponding numbers of cases. Table S2. Distribution of population weighted concentrations of ambient PM2.5 and ozone in Quebec. SD: standard deviation. [file 13075_2022_2843_MOESM1_ESM.docx]

**Supporting Materials**

Table S1. International Classification of Diseases (ICD) diagnostic codes for defining systemic autoimmune rheumatic diseases cases and their corresponding numbers of cases.

| Code | Disease | The number of patients (percentage) |
| --- | --- | --- |
| ICD-9 446 | Polyarteritis nodosa | 735 (21.2) |
| ICD-9 710 | Diffuse diseases of connective tissue | 1220 (35.1) |
| ICD-9 725 | polymyalgia rheumatica | 887 (25.5) |
| ICD-10 M30 | Polyarteritis nodosa | 21 (0.6) |
| ICD-10 M31 | Other necrotizing vasculopathies | 107 (3.1) |
| ICD-10 M32.1 | Systemic lupus erythematosus with organ or system involvement | 7 (0.2) |
| ICD-10 M32.8 | Other forms of Systemic lupus erythematosus | 3 (0.1) |
| ICD-10 M32.9 | Systemic lupus erythematosus, unspecified | 17 (0.5) |
| ICD-10 M33 | Dermatopolymyositis | 2 (0.1) |
| ICD-10 M34 | Systemic sclerosis | 16 (0.5) |
| ICD-10 M35.0 | Sjogren syndrome | 23 (0.7) |
| ICD-10 M35.3 | Polymyalgia rheumatica | 364 (10.5) |
| ICD-10 M35.5 | Multifocal fibrosclerosis | 0 (0.00) |
| ICD-10 M35.9 | Systemic involvement of connective tissue, unspecified | 15 (0.4) |

Table S2. Distribution of population weighted concentrations of ambient PM_2.5_ and ozone in Quebec. SD: standard deviation

| **Year** | **Mean** | **SD** | **Minimum** | **10%** | **25%** | **Median** | **75%** | **90%** | **Maximum** |
| --- | --- | --- | --- | --- | --- | --- | --- | --- | --- |
| PM_2.5_ (μg/m^3^) | | | | | | | | | |
| 2000 | 8.93 | 2.77 | 0.73 | 4.90 | 6.90 | 9.40 | 11.10 | 12.03 | 18.17 |
| 2001 | 9.08 | 2.80 | 1.20 | 5.00 | 6.97 | 9.47 | 11.33 | 12.43 | 18.60 |
| 2002 | 8.95 | 2.75 | 1.20 | 4.97 | 6.83 | 9.17 | 11.23 | 12.37 | 17.53 |
| 2003 | 8.59 | 2.65 | 1.47 | 4.83 | 6.50 | 8.77 | 11.00 | 11.80 | 14.60 |
| 2004 | 8.00 | 2.49 | 1.37 | 4.60 | 5.97 | 8.17 | 10.43 | 10.97 | 13.43 |
| 2005 | 7.48 | 2.32 | 1.37 | 4.40 | 5.50 | 7.60 | 9.80 | 10.27 | 12.73 |
| 2006 | 7.07 | 2.16 | 1.33 | 4.20 | 5.23 | 7.17 | 9.23 | 9.70 | 11.30 |
| 2007 | 6.80 | 2.12 | 1.47 | 4.00 | 5.00 | 6.83 | 8.97 | 9.43 | 11.23 |
| 2008 | 6.63 | 2.07 | 1.30 | 3.87 | 4.87 | 6.70 | 8.73 | 9.17 | 11.93 |
| 2009 | 6.60 | 2.05 | 1.10 | 3.87 | 4.87 | 6.70 | 8.63 | 9.10 | 13.07 |
| 2010 | 6.59 | 2.03 | 0.35 | 3.90 | 4.90 | 6.70 | 8.55 | 9.00 | 14.20 |
| 2011 | 6.65 | 2.05 | 0.30 | 3.90 | 5.00 | 6.80 | 8.60 | 9.10 | 14.20 |
| 2012 | 6.64 | 2.05 | 0.90 | 3.90 | 5.00 | 6.70 | 8.60 | 9.10 | 14.20 |
| Average | 7.54 | 2.55 | 0.30 | 4.20 | 5.40 | 7.63 | 9.37 | 10.93 | 18.6 |
| Ozone (ppb) | | | | | | | | | |
| 2002 | 21.56 | 2.83 | 6.28 | 18.81 | 19.68 | 21.61 | 23.30 | 25.25 | 27.94 |
| 2003 | 22.40 | 3.23 | 13.63 | 18.22 | 19.45 | 22.86 | 25.18 | 26.67 | 30.86 |
| 2004 | 22.66 | 3.44 | 12.05 | 18.52 | 19.18 | 23.06 | 25.65 | 27.70 | 30.91 |
| 2005 | 23.57 | 3.40 | 15.33 | 18.79 | 20.49 | 23.61 | 26.50 | 28.11 | 32.98 |
| 2006 | 22.36 | 2.92 | 17.10 | 18.29 | 20.36 | 22.68 | 24.33 | 26.11 | 30.57 |
| 2007 | 23.38 | 2.71 | 9.82 | 19.87 | 20.69 | 23.59 | 25.32 | 26.76 | 31.19 |
| 2008 | 23.77 | 2.67 | 8.40 | 20.83 | 21.01 | 24.02 | 25.93 | 26.99 | 31.46 |
| 2009 | 24.46 | 2.66 | 9.63 | 21.90 | 22.00 | 24.08 | 26.62 | 27.85 | 31.51 |
| 2010 | 23.90 | 3.01 | 17.69 | 20.43 | 21.17 | 24.14 | 26.44 | 27.81 | 32.04 |
| 2011 | 24.64 | 3.35 | 19.08 | 19.65 | 22.33 | 25.08 | 27.22 | 28.68 | 33.61 |
| 2012 | 31.76 | 3.80 | 24.20 | 27.29 | 27.62 | 32.16 | 34.90 | 36.85 | 40.06 |
| Average | 24.05 | 4.06 | 13.29 | 19.19 | 21.03 | 23.90 | 26.49 | 28.48 | 32.77 |
